# Impact of processing-latency induced interaural delay and level discrepancy on sensitivity to interaural level differences in cochlear implant users

**DOI:** 10.1007/s00405-023-08013-w

**Published:** 2023-05-23

**Authors:** Monika Körtje, Timo Stöver, Uwe Baumann, Tobias Weissgerber

**Affiliations:** 1 ENT Department, Audiological Acoustics, Goethe University Frankfurt, University Hospital Frankfurt, Theodor-Stern-Kai 7, 60590 Frankfurt (Main), Germany; 2 ENT Department, Goethe University Frankfurt, University Hospital Frankfurt, Frankfurt (Main), Germany

**Keywords:** Interaural delay, Interaural level difference, Cochlear implant, Single-sided deafness, Sound localization

## Abstract

**Purpose:**

This study investigated whether an interaural delay, e.g. caused by the processing latency of a hearing device, can affect sensitivity to interaural level differences (ILDs) in normal hearing subjects or cochlear implant (CI) users with contralateral normal hearing (SSD-CI).

**Methods:**

Sensitivity to ILD was measured in 10 SSD-CI subjects and in 24 normal hearing subjects. The stimulus was a noise burst presented via headphones and via a direct cable connection (CI). ILD sensitivity was measured for different interaural delays in the range induced by hearing devices. ILD sensitivity was correlated with results obtained in a sound localization task using seven loudspeakers in the frontal horizontal plane.

**Results:**

In the normal hearing subjects the sensitivity to interaural level differences deteriorated significantly with increasing interaural delays. In the CI group, no significant effect of interaural delays on ILD sensitivity was found. The NH subjects were significantly more sensitive to ILDs. The mean localization error in the CI group was 10.8° higher than in the normal hearing group. No correlation between sound localization ability and ILD sensitivity was found.

**Conclusion:**

Interaural delays influence the perception of ILDs. For normal hearing subjects a significant decrement in sensitivity to ILD was measured. The effect could not be confirmed in the tested SSD-CI group, probably due to a small subject group with large variations. The temporal matching of the two sides may be beneficial for ILD processing and thus sound localization for CI patients. However, further studies are needed for verification.

## Introduction

Patients supplied with either one or two cochlear implants (CIs) have limited access to monaural and binaural auditory cues compared to normal hearing (NH) listeners and, therefore, difficulties in localizing sounds. However, CI users with bilateral CIs or unilateral CI using a hearing aid on the contralateral ear (bimodal) are also able to localize sounds, but less precisely than NH or CI users with single-sided deafness (SSD) [1–3].

The most relevant cues for sound localization are interaural time differences (ITD) and interaural level differences (ILD). Sensitivities to ITDs and ILDs are highly correlated and part of a complex neural processing [4, 5]. Deteriorated ITD and ILD sensitivity occurs even with mild hearing loss [5, 6]. In NH subjects, low frequency ITD information dominates when available [4, 7], whereas CI users primarily use ILD information for sound localization [1, 8, 9]. In 2018, Prejban et al. described that SSD-CI subjects were able to lateralize sounds with ILDs presented via headphones [10].

In SSD-CI or bimodally fitted CI users, the different auditory inputs of both ears (acoustic vs. electric stimulation) diminish binaural processing [11]. For example, a hearing aid and a CI have different processing times until the auditory nerve is stimulated. In a study by Zirn and coworkers, it was shown that the time period from signal presentation to stimulation of the auditory nerve with a CI (manufacturer MED-EL, Innsbruck, Austria) is comparable to that of an NH ear for stimulation frequencies around 1 kHz [12]. Higher frequencies arrive earlier in the NH ear than with electric stimulation and lower frequencies arrive later in the NH ear, due to the mechanics of the traveling wave effect in the cochlea. Processing delays of other CI manufacturers are frequency-independent and in the range of 9 to 12.5 ms [13] and most hearing aids have processing delays between 3 and 11 ms [14]. Recent studies showed that sound localization can be improved by minimizing the interaural delays between electrical CI stimulation and acoustic stimulation by a hearing aid [15, 16]. The best results in sound localization of speech-shaped signals were achieved in SSD-CI subjects with a small additional delay of about 1 ms on the CI side [17].

However, it is still unclear, which binaural cue is improved by the temporal matching of the two sides. One hypothesis is that the adjusted temporal signals on both sides allow processing of more synchronized ITD information. However, since access to ITD cues is only limited in CI systems, one other hypothesis is that the better timing is beneficial for more accurate temporal integration of ILD cues. The effect of interaural delay on sensitivity to ILD cues has not been adequately investigated so far, even in NH subjects.

The aim of this study was to measure to which extend interaural processing delays affect ILD sensitivity in NH subjects or experienced SSD-CI subjects. For this purpose, the sensitivity to ILDs was determined and compared for different interaural delays. In addition, sound localization abilities were measured in both subject groups and results were compared with the results of ILD sensitivity.

## Materials and methods

### Subjects

10 SSD-CI users (mean age: 51 years, 6 female and 4 male) and 24 NH subjects (mean age: 28 years, 16 female and 8 male) participated in this study.

**Fig. 1:**
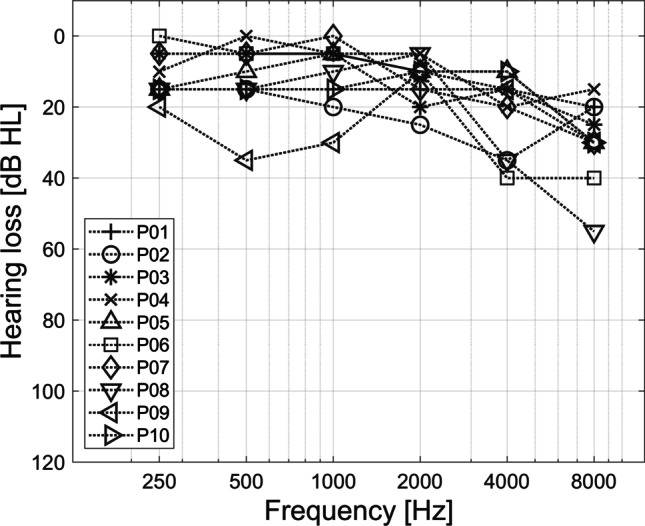
Pure-tone audiogram thresholds (air conduction) of the normal hearing ear of the SSDCI subjects

Only SSD-CI subjects with a mean contralateral pure-tone audiogram thresholds (pure-tone average PTA-4 of 0.5, 1, 2 and 4 kHz) better or equal 25 dB HL and without contralateral hearing aid were included in the study. The mean PTA-4 was 14.3 ± 5.5 dB HL. Individual pure-tone audiograms are visualized in Fig. 1. The demographic data of the SSD-CI subjects is shown in Table 1.

**Table 1:** Demographic data of SSD-CI subjects

Code	Age [years]	Device	CI experience [years]	Duration of hearing loss* [years]	FMS CI [%]	PTA-4 [dB HL]
P01	52	CONCERTO	4	1	45	15
P02	58	CONCERTO	5	10	40	24
P03	54	CONCERTO	4	20	25	11
P04	49	SYNCHRONY	3	10	70	8
P05	37	CONCERTO	5	0	25	9
P06	59	SYNCHRONY	4	1	65	15
P07	48	SYNCHRONY	1	5	35	10
P08	77	CONCERTO	6	3	60	16
P09	37	SYNCHRONY	1	4	20	23
P10	40	SYNCHRONY	3	0	40	13
mean	51		3.6	5.4	42.5	14.3

*FMS* Freiburger monosyllables

^*^Corresponds in 7 of 10 SSD-CI subjects to duration of deafness. For 3 subjects duration of deafness is unknown

All SSD-CI participants received implants from the same manufacturer (MED-EL, Innsbruck, Austria) with Flex28 electrode arrays and used the stimulation strategy encoding temporal fine structure in the four most apical electrodes FS4. All subjects used behind the ear processors (OPUS2 or SONNET) and had at least one year of experience with their CI. The mean monosyllable speech perception score was 42.5% at 65 dB sound pressure level (Freiburg monosyllable score, FMS [18]). Measurements were made in a free field condition with contralateral masking (insert earphones, broadband noise of 60 dB SPL).

All NH subjects had a PTA-4 better than 15 dB HL and hearing thresholds lower than 30 dB HL for all octave frequencies between 250 Hz and 8 kHz. The individual interaural difference in PTA-4 was 10 dB HL or less. All SSD-CI subjects suffered from postlingual sensorineural hearing loss prior to CI implantation. In six subjects, deafness was caused by one or more sudden hearing losses, two suffered from Menière’s disease, one from head trauma and one from otitis media affecting the inner ear.

The study was approved by the local institutional review board (No. 213/16). All subjects gave their written consent and the SSD-CI subjects received financial compensation for participating in this study. The tests were conducted in accordance with the Code of Ethics of the World Medical Association (Declaration of Helsinki) for experiments with humans.

### Loudness balancing for SSD-CI subjects

Prior to the ILD and localization task, the SSD-CI subjects had to perform an interaural loudness-balancing to match the perceived loudness in the CI ear with the contralateral ear. To ensure sufficient dynamic range, the volume level of the CI processor in the subject’s everyday program was set to at least 85% in the Maestro fitting software (MED-EL, Innsbruck, Austria). If the currently used volume level was below 85%, the most comfortable level (MCL) was lowered globally and the volume level was increased afterwards.

The stimuli in the CI ear were presented via auxiliary input of the sound processor driven by a headphone amp (Lake People, Konstanz, Germany) equipped with an analog volume potentiometer. The stimuli in the contralateral ear were presented using a headphone (Sennheiser HDA 200, Wedemark, Germany) driven by the integrated headphone-amp of the used audio interface and digital to analog converter RME Fireface UC (Audio AG, Haimhausen, Germany) and calibrated to a fixed sound pressure level of 65 dB SPL. All tasks were implemented with software MATLAB R2010a (MathWorks, Natick, United States).

The used stimulus was a broadband noise burst (bandpass filtered between 100 Hz and 15 kHz) with a duration of 500 ms and a time ramp of 20 ms. The stimulus was presented twice to the left ear and then twice to the right ear. Pauses in between stimuli were 200 ms. The subjects were asked whether the perceived loudness were the same on both sides. If one side was perceived louder, level was adjusted on the CI-side by the experimenter and the test was repeated unlimited times until a loudness considered as equal on both sides was reached. Afterwards, the loudness balance was rechecked with the same stimulus alternating between the left and right ear to obtain the final setting and potentially corrected again in level by the subject with the remote control until a loudness considered as equal was reached.

### ILD sensitivity (just noticeable difference)

All tests were performed in an anechoic chamber (size: 4.1 × 2.6 × 2.1 m, reverberation time RT60: 0.05 s). The just noticeable differences (JND) of ILDs were measured using a two alternative forced choice method (2-AFC) with a 1up-2down-procedure to estimate the threshold of 70.7% correct [19]. The task was realized with the psylab-toolbox (version 2.8, Jade Hochschule Oldenburg, Germany, [20]). The initial ILD was 15 dB with a step size of 6 dB. The step size was halved after each reversal and the minimum step size was 0.75 dB. The test stopped after six reversals with minimum step size. A reversal is defined as local minimum or local maximum. The mean of the local minimum and maximum values of the JND task with minimum step size was defined as JND-ILD.

Two consecutive pairs of sound stimuli were presented dichotically to both ears. One pair had fixed ILDs and one pair had adaptively changing ILDs per trial. The order of the pairs was randomized. The subjects were asked to indicate the change in perceived sound position between the two signals (left–right or right-left). A level roving of ± 5 dB was implemented to avoid any monaural intensity cues.

To keep the perceived binaural loudness for each tested ILD constant, the level adjustments for each ear was based on the level calculation of an ideal panpot of a mixing desk [21]. ILD cues were presented as an asymmetrical level separation of the ILD information by increasing the level on one side by a smaller amount and decreasing the level of the other side by a larger amount. In the group of NH subjects, the signal was always lateralized towards the left ear, i.e. signals with ILDs had a higher level on the left side than on the right side. For the SSD-CI group the signal was always lateralized towards the normal hearing ear.

Each subject performed 8 trials with different test stimuli. Two different reference ILDs were tested (0 dB and 10 dB) with 4 different interaural delays each. For the reference ILD of 10 dB, the level on the left ear (or normal hearing ear in SSD-CI subjects) was increased by 5 dB, whereas the right ear (or CI in SSD-CI subjects) was decreased by 5 dB. To realize the interaural delay, the left ear in NH and the normal-hearing ear in SSD-CI subjects was delayed. For the NH subjects delays of 0, 5, 10 and 15 ms were tested to cover the range of processing delays potentially introduced by hearing aids or CIs [14]. The SSD-CI subjects were tested with delays of -5, 0, 5 and 10 ms, whereas 5 ms refers to a delay of the CI ear. Each subject completed an initial training trial with a reference ILD of 0 dB and no interaural delay.

### Sound localization test

The sound localization was tested for seven frontal loudspeaker positions within ± 60° (0°, ± 21°, ± 42°, ± 59°). Each position was tested five times in random order. The sound stimuli were five pulsed broadband noise bursts (length 30 ms, 3 ms ramping, pause 70 ms), presented at 65 dB SPL with a level roving of ± 6 dB. The subjects were asked to fixate an initial active LED at 0° during stimulus presentation to avoid cues from head movements. The subjects indicated the perceived position by changing the active LED of a frontal LED array with a rotary encoder. The loudspeakers were hidden behind an acoustically transparent curtain to avoid visual cues. No feedback was given to the subjects during the test. Prior to testing, a training session was conducted to assure the correct handling. The setup is further described in earlier studies [22]. One SSD-CI subject (P10) and two NH subjects did not perform the sound localization task and are therefore excluded from the following analysis.

The localization error was defined as the difference between the perceived and the presented angle. To obtain a single value for localization accuracy, the mean localization error (MLE) was calculated as the median of the localization errors per angle. Additionally, the interquartile ranges per angle were calculated to estimate the uncertainty of sound localization.

### Statistics

Boxplots and median values were used for descriptive analyses throughout the manuscript. Nonparametric tests were utilized for statistical analyses of ILD sensitivity and the sound localization accuracy. The Mann Whitney U-test was applied for pairwise group comparisons between SSD-CI subjects and NH. Statistical differences of JND-ILDs within the subject groups were determined with the Friedman test. Correlations were tested via Spearman rank correlation. A p-value < 0.05 was considered as significant and a Bonferroni correction of the p-value was performed in case of multiple pairwise comparison. IBM SPSS Statistics 27 (IBM, Armonik, New York) was used for the analysis.

## Results

### JND-ILD

**Fig. 2:**
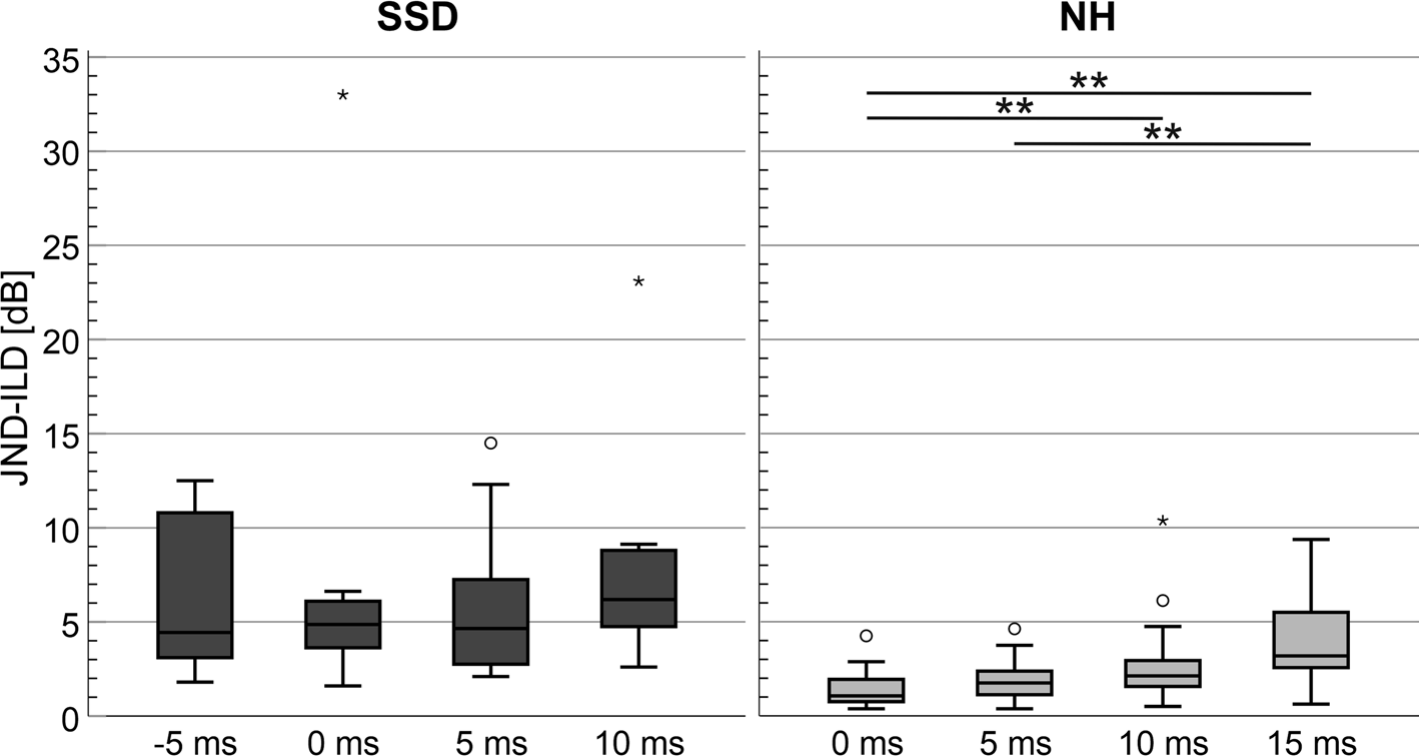
Boxplots of JND-ILD of SSD-CI subjects (left) and normal hearing subjects (right) for a reference ILD of 0 dB with four different interaural delays

All subjects were able to perform the test and to perceive a lateralized sound impression when ILD cues were presented. The JND-ILDs (reference ILD: 0 dB) for both subject groups are plotted in Fig. 2. The median JND-ILDs in the SSD-CI group varied from 4.9 dB (no delay) to 6.2 dB (10 ms delay). No significant effect of interaural delay on JND-ILDs was found in this subject group (*Z* = 1.788, *p* = 0.618). The median JND-ILD in the NH group varied between 1.1 dB (no delay) and 3.2 dB (15 ms delay). The JND-ILDs were significantly affected by an interaural delay at a reference ILD of 0 dB (*Z* = 40.190, *p* < 0.001). In a paired post-hoc comparison, significant differences between JND-ILDs were found between 0 and 10 ms (*Z* = -3.410, *p* = 0.004), 0 and 15 ms (*Z* = -5.981, *p* < 0.001) and between 5 and 15 ms (*Z* = -4.416, *p* < 0.001).

The NH subjects performed significantly better in all conditions (0 ms: *Z* = 4.202, *p* < 0.001; 5 ms: *Z* = 3.559, *p* < 0.001; 10 ms: *Z* = 3.576, *p* < 0.001). A few SSD-CI subjects yielded ILD sensitivities, which were comparable to NH subjects.

**Fig. 3:**
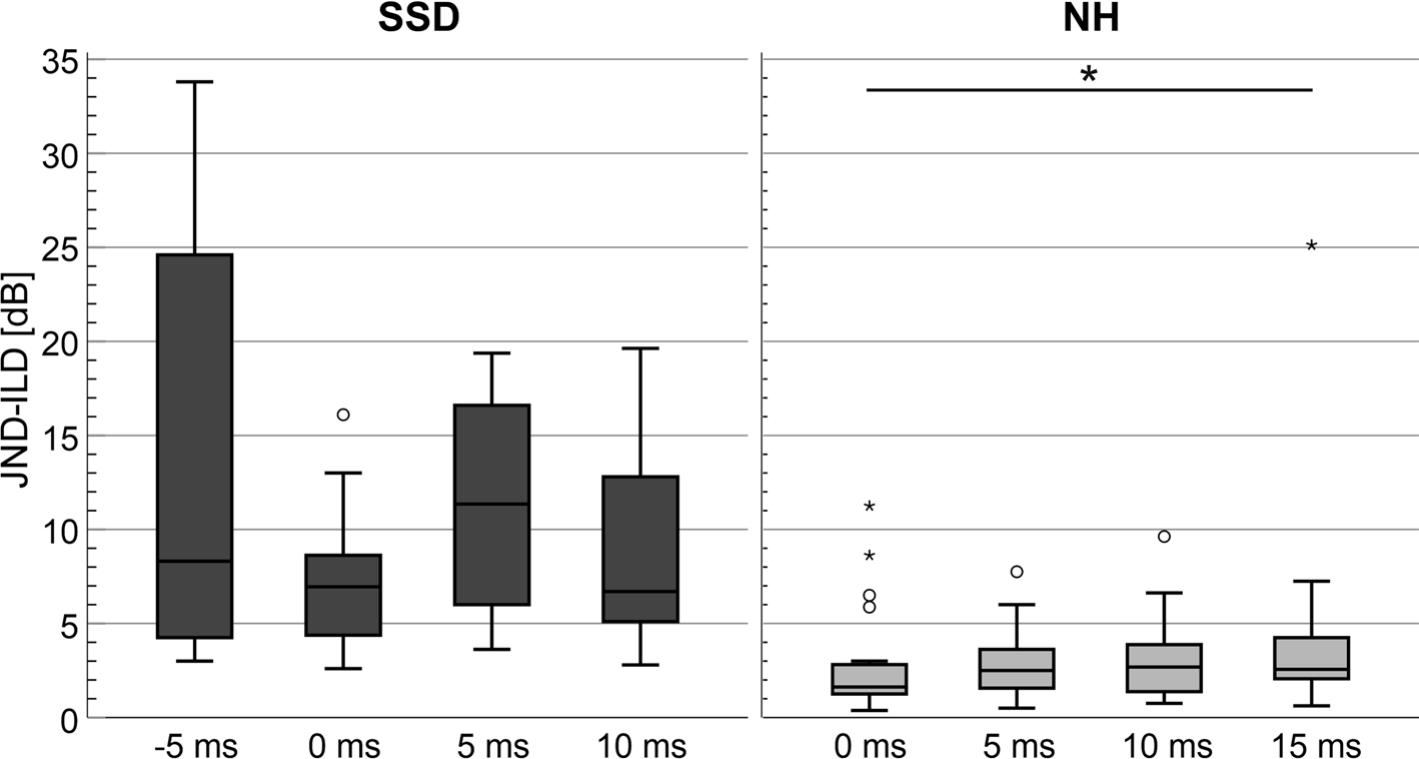
Boxplots of JND-ILDs for SSD-CI and NH subjects for a reference ILD of 10 dB with four different interaural delays. Data points of two outliers are not shown for a better readability of the graph: P08(-5 ms): 57.25 dB; P08(5 ms) = 43.5 dB

The results for a reference ILD of 10 dB are visualized in Fig. 3. The median JND-ILDs in the SSD-CI group varied from 6.7 dB (10 ms delay) to 11.4 dB (5 ms delay). No impact of interaural delay on JND-ILDs was found in this subject group (*Z* = 6.455, *p* = 0.091). The median JND-ILD in the NH group varied between 1.6 dB (no delay) and 2.7 dB (10 ms delay). The JND-ILDs were significantly affected by an interaural delay at a reference ILD of 10 dB (*Z* = 7.826, *p* = 0.050). The JND-ILD with no delay was significantly lower than the JND-ILD for 15 ms delay (*Z* = -2.739, *p* = 0.037). The NH subjects performed significantly better in all conditions (0 ms: *Z* = 3.406, *p* < 0.001; 5 ms: *Z* = 4.027, *p* < 0.001; 10 ms: *Z* = 3.479, *p* < 0.001).

JND-ILDs compared to a reference ILD of 0 dB increased in both subject groups. For the NH subjects, the increase was largest but non-significant for 0 and 5 ms (0 ms: Z = − 2.227, *p* = 0.104; 5 ms: *Z* = − 2.435, *p* = 0.06), for the SSD-CI subjects the increase in JND-ILD was significant for 5 ms (5 ms: *Z* = − 2.803, *p* = 0.02). It should be noted that interquartile ranges in the SSD-CI group considerably increased for the reference ILD of 10 dB compared to 0 dB, especially for a delay of -5 ms.

For all tested interaural delays, no correlation between age and ILD sensitivity was found in both subject groups.

### Sound localization test

**Fig. 4:**
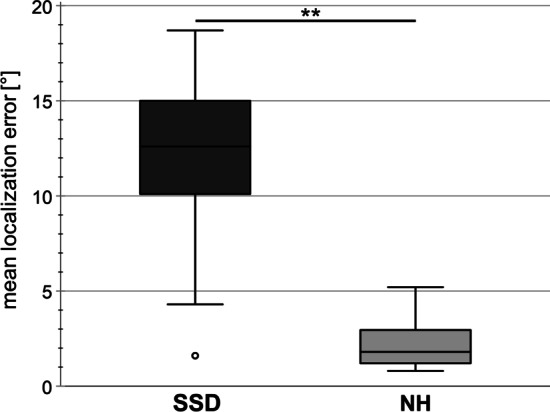
Localization error of SSD-CI and NH subjects

Boxplots of the localization error are shown in Fig. 4. The median localization error was 12.6° for the SSD-CI subjects (range: 1.6–18.7°) and for the NH-subjects the median localization error was 1.8° (range: 0.8–5.2°). The NH subjects performed significantly better than the SSD-CI subjects (*Z* = 3.375, *p* < 0.001). In both subject groups, no correlation was found between the results of the sound localization task and the JND-ILD task with a reference ILD of 0 dB (SSD-CI: *r*_s_ = − 0.200, NH: *r*_s_ = − 0.150) and with a reference ILD of 10 dB (SSD-CI: *r*_s_ = − 0.503, NH: *r*_s_ = 0.297).

**Fig. 5:**
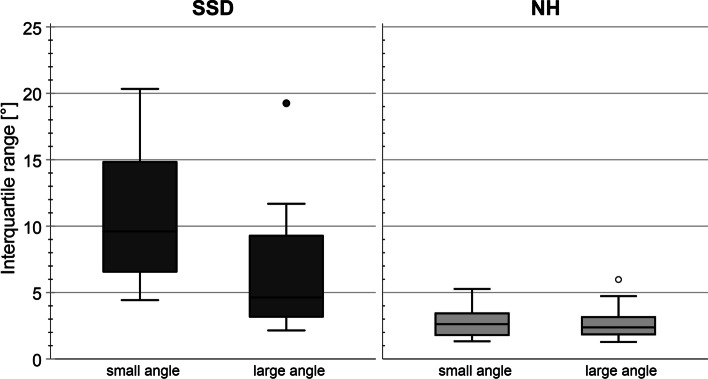
Mean interquartile ranges of sound localization results for small angles (±21°, 0°) and for large angles (±42°, ±58°) for both subject groups

Interquartile ranges (IQR) of localization task were used to analyze the uncertainty of sound localization in the different subject groups. IQR were separately analyzed for more central and smaller (± 21° and 0°) and more lateral and larger (± 42° and ± 59°) angles of sound incidence (Fig. 5). While no difference in IQR was detectable in the NH subjects, the SSD-CI subjects had a more reliable sound localization for the larger angles than for the smaller angles (NH: *Z* = -1.057, *p* = 0.290; SSD-CI: *Z* = − 1.886, *p* = 0.059).

## Discussion

Sensitivity to interaural level differences was measured in 10 SSD-CI subjects and 24 NH subjects for broadband noise pulses presented via headphones and via a direct cable connection into the CI. The impact of interaural delays on ILD sensitivity was investigated for two different reference ILDs. Results were correlated with localization accuracy for frontally presented signals (broadband noise).

NH subjects showed decreasing ILD sensitivity with increasing interaural delay for both reference ILDs. In the SSD-CI group, no significant impact of interaural delay on ILD sensitivity was found, possibly due to the high individual variability in the results. Another aspect could be that generally worse ILD sensitivity in this subject group hinder the detection of the (even in the NH relatively small) JND differences induced by interaural delays. The median JND-ILD for SSD-CI subjects varied between 4.7 and 11.3 dB and was significantly higher than for the NH group. For a reference ILD of 0 dB, some of the SSD-CI subjects were able to achieve ILD sensitivities in the range of NH.

This is the first study to investigate the impact of interaural processing latencies on ILD sensitivity in NH and SSD-CI subjects.

### ILD sensitivity

Although several studies investigating ILD sensitivity in NH subjects were published, there is currently nearly no data available discussing ILD sensitivity in CI users. The results obtained in experiments measuring JNDs for ILD sensitivity are highly dependent on the experimental design (e.g. type of stimulus, test procedure, etc.). Therefore, results of different studies are only comparable to a limited extend. In the present study, a short broadband stimulus was used, whereas some other studies with NH subjects used narrowband noise signals with different center frequencies [23]. In some other studies an AFC method was used [24], whereas in different studies subjects were asked to estimate the lateralized side for signals with defined ILDs [10, 23].

In a study by Ochi et al. in 2014 [24], sensitivity thresholds for ILDs and ITDs were measured in NH subjects for low- and high-frequency signals (duration 400 ms). The JND-ILD ranged from 1 to 4 dB for the low-frequency signals and from 1 to 5 dB for the high-frequency signals [24]. Spencer and colleagues also measured JND-ILDs in NH subjects for low- and high-frequency signals and obtained mean JND-ILDs of 1.9 dB [25], similar to Bernstein and colleagues with a JND-ILD of 2 dB [6]. However, Bernstein et al. used signals with center frequencies of 500 Hz or 4 kHz rather than broadband noise signals. Although the experimental design in our study was completely different, the median JND-ILD in this study of 1.1 dB for NH subjects obtained in our study is in line with previous studies. Since broadband noise was used in our study, this may explain the slightly better ILD sensitivity compared to the results of other studies.

Only a limited number of studies investigated on the ILD sensitivity in SSD-CI subjects so far. In a study published by Prejban and colleagues in 2018 [10] lateralization abilities of SSD-CI subjects based on ILDs were measured for ILDs between 0 and 24 dB with a step size of 4 dB. Since some of the SSD-CI subjects analyzed in their study were able to lateralize signals with ILDs of 4 dB, a sensitivity threshold in the range of 4 dB can be assumed, which is in the range of the results of our study obtained for a reference ILD of 0 dB without interaural delay.

In a study by Francart and colleagues in 2008, JND-ILD was estimated in bimodally fitted subjects by measuring psychometric functions [26]. The estimated median JND-ILD was 2 dB and, thus, better than the results of SSD-CI subjects found in our study with or without interaural delay. However, they stimulated the CI and the hearing aid directly (after conducting a pitch matching procedure) and measured the loudness growth function by loudness balancing both sides for different levels.

The SSD-CI subjects performed significantly worse than NH in all ILD tasks. One possible explanation is the reduced auditory input with a CI combined with a diminished binaural integration of the auditory information. It should also be considered that the acoustic and the electric inputs had different and unmatched dynamic ranges. With an adapted or enhanced electrical dynamic range, as performed by Gajecki and Nogueira 2021, the ILD sensitivity could be improved for CI subjects [27]. Also, different loudness growth functions for acoustic and electric stimulation might hinder better ILD sensitivity in SSD-CI subjects [26]. Another aspect that could account for different results of NH and SSD-CI subjects was the age difference in both subjects groups. The SSD-CI group was significantly older than the NH subject group. Several studies reported declining sensitivity to binaural cues with age [28, 29], but they focused mainly on the temporal integration processing. An early study by Herman et al. in 1977 assumed that age had no effect on level-based lateralization cues, but only on temporal cues [30]. However, because of the small subject groups and age ranges the results of our study do not allow us to draw conclusions about age effects on JND-ILDs.

### Impact of processing delays

While interaural delays decreased ILD sensitivity in NH subjects, no significant effects were found in SSD-CI subjects. NH results are in line with a study from 2015 from Kong et al., where detrimental effect of interaural delays on binaural processing was found [31]. Angermeier and colleagues reported recently that an additional interaural delay deteriorates speech perception in noise and decreases spatial release from masking [32]. They measured speech perception in noise with varying interaural delays from 0 to 7 ms and were obtained in NH subjects.

To the authors’ knowledge, the impact of interaural delays on ILD sensitivity in CI subjects was not investigated so far. Some previous studies measured the effects of interaural delays on sound localization accuracy in SSD-CI users [17, 33]. Zirn and coworkers [33] delayed the CI to compensate for the hearing aid delay in bimodally fitted subjects, whereas Seebacher et al. [17] investigated whether a small additional delay of the CI (delay values below 4 ms) could improve the localization accuracy. In both studies, the temporal matching of both sides resulted in improved localization accuracy. Another study found no benefit in sound localization by matching the CI to the hearing aid delay [2]. In our study no significant correlation was found between JND-ILD and sound localization errors. However, in our study a large range of processing delays with rather large step size was tested in the JND task.

In our study, no significant impact of the interaural delay on the ILD sensitivity was found for the SSD-CI subjects. However, trend towards decreased ILD sensitivity for higher interaural delays were shown. Potential factors that may have reduced an effect were the small sample of subjects and the large inter-individual variances in JND-ILD results. It is also possible that the high compression of CI stimulation minimizes sensitivity to ILDs. Based on the frequency-specific delays in CI measured by Zirn and coworkers, it may be relevant to compensate the interaural delay in a frequency-specific manner [11, 12].

### Correlation to localization accuracy

No correlation was found between JND-ILD and localization accuracy in both subject groups. In the NH group, this might be explained by the very good (i.e. low) JND-ILD for all subjects. The differences in ILD between the seven tested loudspeakers is higher than measured JND-ILD. Furthermore, NH subjects are relying predominantly on ITD cues, which are also present in the broadband noise of the localization task.

In the SSD-CI group, ILD information is shown to be dominant for sound localization since high-pass and broadband signals can be localized more reliably than low pass signals [1]. Due to the individual variability in JND-ILDs, a correlation with sound localization ability could be assumed. However, no correlation was found. A different explanation could be the measurement method used for sound localization. The results of sound localization are highly dependent on the experimental task and the used method of the analysis of the results. In our study, the mean localization error was used which is a single measure for sound localization averaged over all tested angles of sound incidence. A different measure is the root mean square (RMS) value. In a study reporting the RMS value, localization abilities in bilateral CI users were described with a mean RMS value of 20.4° [9]. Furthermore, since the difference of angular placement of tested loudspeakers was quite high, high ILD cues were available in the localization task. In the SSD-CI group it was shown in the analysis of interquartile ranges of the localization results, that these were more certain in the identification of the position of (i.e. higher ILDs) of sound incidence compared to lower ILDs. For a potential correlation with ILD sensitivity, it would be beneficial to test for a minimum audible angle instead of localization ability for different loudspeaker positions.

Another potential explanation for the lack of correlation is the small number of SSD-CI subjects in this study. Further studies are necessary to investigate the relation between sound localization and ILD sensitivity in CI users.

## Conclusion

A detrimental effect of interaural delay on ILD sensitivity was found in NH. The effect could not be confirmed in the tested SSD-CI group. Based on this result, the beneficial effects of binaural temporal matching shown in other studies could not be explained by improved ILD sensitivity.

## Data Availability

Data available on request from the authors.
